# *De Novo* Biosynthesis of Curcumin in *Saccharomyces cerevisiae*

**DOI:** 10.1021/acssynbio.4c00059

**Published:** 2024-05-24

**Authors:** João Rainha, Joana L. Rodrigues, Lígia R. Rodrigues

**Affiliations:** † Centre of Biological Engineering, University of Minho, Braga 4710-057, Portugal; ‡ LABBELS—Associate Laboratory, Braga 4710-057, Portugal

**Keywords:** curcumin biosynthesis, Saccharomyces cerevisiae, *de novo* production, CRISPR-Cas9, synthetic biology

## Abstract

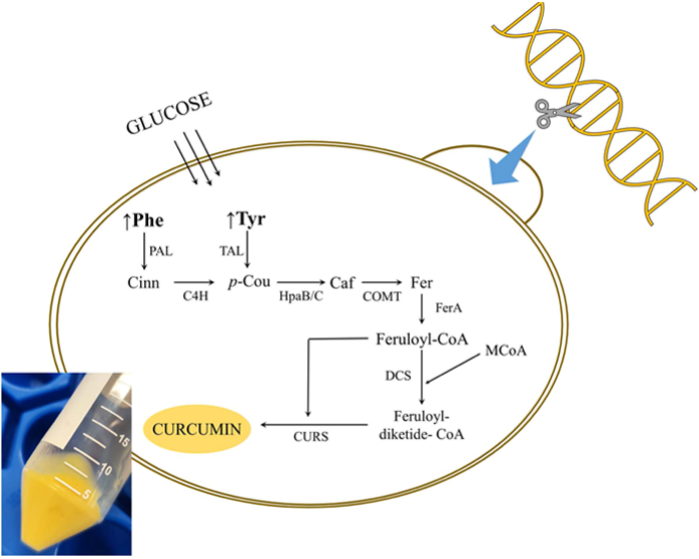

Curcumin, a natural polyphenol derived from turmeric, has attracted immense interest due to its diverse pharmacological properties. Traditional extraction methods from *Curcuma longa* plants present limitations in meeting the growing demand for this bioactive compound, giving significance to its production by genetically modified microorganisms. Herein, we have developed an engineered *Saccharomyces cerevisiae* to produce curcumin from glucose. A pathway composed of the 4-hydroxyphenylacetate 3-monooxygenase oxygenase complex from *Pseudomonas aeruginosa* and *Salmonella enterica*, caffeic acid *O*-methyltransferase from *Arabidopsis thaliana*, feruloyl-CoA synthetase from *Pseudomonas paucimobilis*, and diketide-CoA synthase and curcumin synthase from *C. longa* was introduced in a *p-*coumaric acid overproducing *S. cerevisiae* strain. This strain produced 240.1 ± 15.1 μg/L of curcumin. Following optimization of phenylpropanoids conversion, a strain capable of producing 4.2 ± 0.6 mg/L was obtained. This study reports for the first time the successful *de novo* production of curcumin in *S. cerevisiae*.

## 1 Introduction

Curcumin, a natural yellow-orange phenolic compound found in turmeric (*Curcuma longa*), has recently gained recognition due to its potential as a cancer-fighting drug.^1^ However, curcumin’s versatility extends beyond its potential as a cancer-fighting agent. Preclinical trials have unveiled a spectrum of additional biological and therapeutic activities due to their anti-inflammatory and antioxidant activities.^2,3^ These findings open opportunities to explore curcumin’s potential for a wide range of health-related issues, making it an exciting candidate for further research and development in the field of natural medicine. Numerous dietary supplements containing curcumin are derived from raw turmeric rhizomes, which may contain impurities and do not provide a pure representation of curcumin.^4^ Several conditions can affect the curcumin content of turmeric rhizomes that ranges from 2 to 5% of curcumin by weight.^5^ Curcumin extraction is usually accomplished using organic solvents and high temperatures such as in Soxhlet extraction.^6^ Turmeric extracts usually contain a mixture of three curcuminoids, with curcumin being the most representative (∼75%), followed by demethoxycurcumin (∼15%) and bisdemethoxycurcumin (∼5%).^7^ Pure curcumin can be obtained from a curcuminoid extract using purification techniques like chromatography procedures.^8^ In summary, the process of extracting and purifying curcumin from plants is quite laborious. Moreover, for large-scale production, curcumin plant extraction is limited by plant seasonality and the large costs and sustainability problems associated with plant crops. On the other hand, curcumin can be chemically synthesized using acetylacetone and boron trioxide in a very complex and harsh process.^9^ A promising biotechnological solution for the high-level production of curcumin involves fermentation using genetically engineered microorganisms. Microorganisms have fast production cycles and can grow in inexpensive substrates, surpassing the limitations of traditional curcumin production methods. The microbial production of curcuminoids has been achieved in *Escherichia coli*,^10−16^*Saccharomyces cerevisiae*,^17^ and in other less conventional hosts such as *Pseudomonas putida*, *Aspergillus oryza*, and *Yarrowia lipolytica*.^18−20^ However, most of these works involve the supplementation of costly curcumin precursors, which is a limitation for potential industrial applications. The *de novo* synthesis of curcumin in microbes offers an attractive and cost-effective solution for scalability. However, curcumin has only been synthesized from simple carbon sources in genetically modified *E. coli*.^12,21^*E. coli* is not a Generally Regarded As Safe (GRAS) organism, and the expression of the heterologous genes usually relies on the use of plasmids, which require constant selective antibiotic pressure for maintaining, and the supplementation of expensive inductors, which is also not desirable. The yeast *S. cerevisiae* is a role model microorganism for genetic engineering purposes due to its safety status and clear metabolic pathways. The extensive molecular tools available, such as the Clustered Regularly Interspaced Palindromic Repeats (CRISPR)-Cas9 based ones, allow the genomic integration of multiple heterologous genes without requiring antibiotic markers.^22^ Furthermore, significant research has been dedicated to the biosynthesis of plant polyphenols in this yeast.^23,24^ Curcumin biosynthesis, in *C. longa*, is accomplished by reactions catalyzed by the type III polyketide synthases (PKSs), diketide-CoA synthase (DCS), and curcumin synthase (CURS). Three isoforms of CURS were identified in *C. longa*, each one with a different substrate affinity explaining the diversity of curcuminoids found in turmeric.^25^ The phenylpropanoids *p*-coumaric and/or ferulic acid are precursors of curcuminoids. In the case of curcumin, two molecules of ferulic acid are required, while for demethoxycurcumin, for instance, a ferulic acid and a *p*-coumaric acid molecule are used. First, phenylpropanoids are activated by condensation with a coenzyme A (CoA) molecule, a reaction carried by 4-coumarate-CoA ligase (4CL). After that, DCS adds a malonyl group to the activated phenylpropanoid through a reaction with an extender molecule, the malonyl-CoA. Afterward, CURS catalyzes the curcuminoid synthesis by condensation of the extended activated phenylpropanoid with another activated phenylpropanoid molecule.^26^ The phenylpropanoids are synthesized from the aromatic amino acids, phenylalanine and tyrosine, via the phenylpropanoid pathway, which involves the reactions catalyzed by tyrosine ammonia lyase (TAL), phenylalanine ammonia lyase (PAL), *trans*-cinnamate 4-monooxygenase (C4H), 4-coumarate 3-hydroxylase (C3H), and caffeic acid *O*-methyltransferase (COMT) and has cinnamic acid, *p*-coumaric acid, caffeic acid, and ferulic acid as intermediates.^27^

In recent years, several studies have reported *de novo* high-level biosynthesis of curcumin phenylpropanoid intermediates in yeast, including *p*-coumaric acid,^28^ caffeic acid, and ferulic acid,^29^ identifying heterologous enzymes with optimal performance and endogenous modifications to enhance production yields. In prior research, we examined engineered *S. cerevisiae* capability to produce curcumin by supplementing ferulic acid.^17^ In this study, our goal was to accomplish curcumin *de novo* biosynthesis, eliminating the need for precursor supplementation. In addition, our previous approach involved the use of a plasmid for gene expression, which is not conducive to large-scale applications. To address this, we herein integrated curcumin biosynthesis genes into the yeast genome using a CRISPR-Cas9 approach. In summary, we explored metabolic engineering strategies with the aim of developing an efficient cell factory for curcumin production.

## 2 Results and Discussion

### 2.1 Biosynthesis of Curcumin from Supplemented Substrates

**Figure 1:**
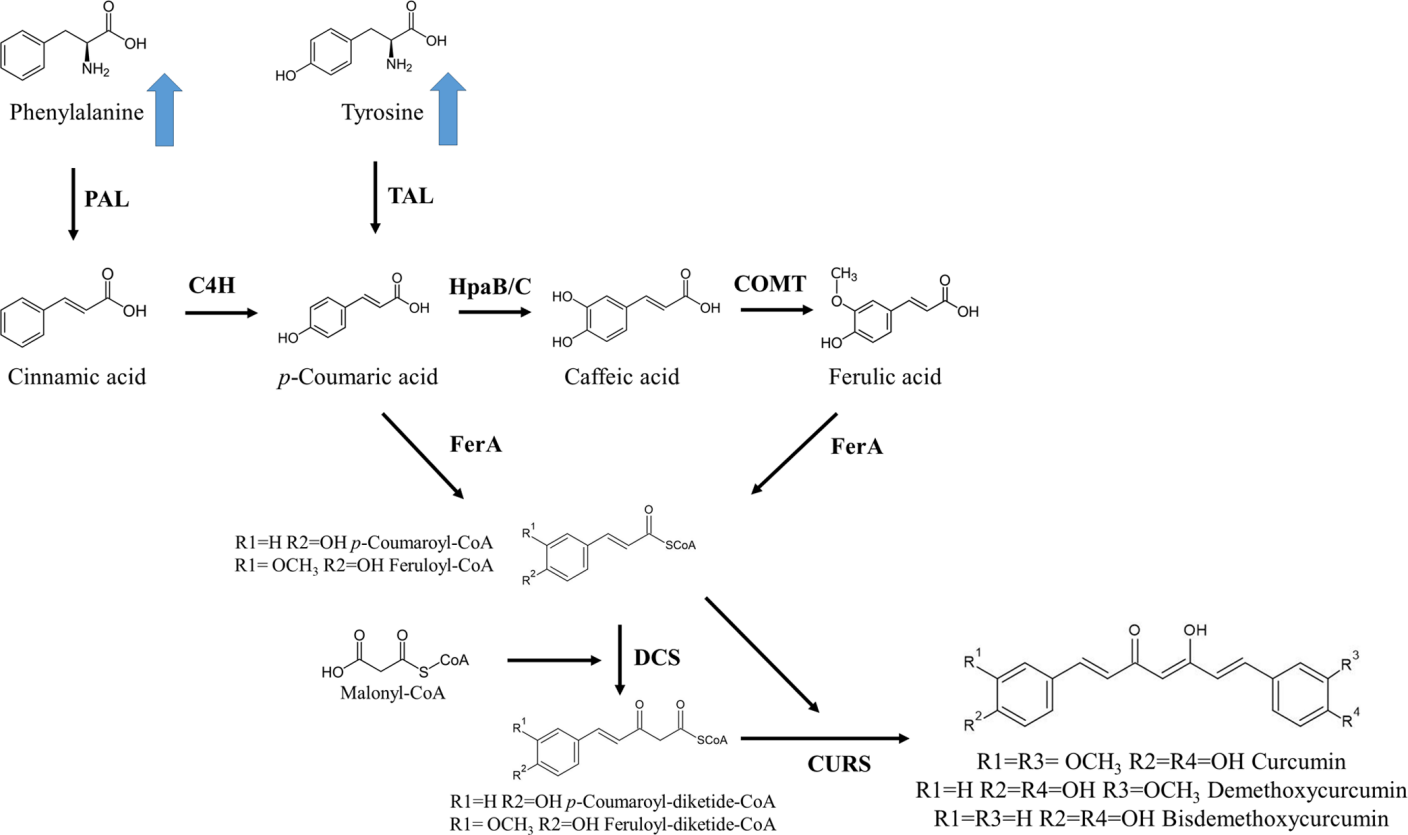
Schematic representation of the artificial biosynthetic pathway constructed for de novo curcumin biosynthesis in *S. cerevisiae*. Blue arrows indicate that the strain is overproducing aromatic amino acids. Enzymes names: PAL: Phenylalanine ammonia lyase; TAL: Tyrosine ammonia lyase; C4H: *Trans*-cinnamate 4-monooxygenase; HpaB/C: 4-hydroxyphenylacetate 3-monooxygenase oxygenase and reductase components; COMT: Caffeic acid *O*-methyltransferase; FerA: feruloyl-CoA synthetase; DCS: Diketide-CoA synthase; and CURS1: Curcumin synthase 1.

In our previous study,^17^ we compared the efficiency of different pathways to synthesize curcumin in *S. cerevisiae* from supplemented ferulic acid. A pathway composed of feruloyl-CoA synthetase (FerA) from *Pseudomonas paucimobilis* (*Pp*FerA) and type III PKS DCS and CURS from *C. longa* (*Cl*DCS and *Cl*CURS) (Figure 1), achieved the highest curcumin titer relative to other enzyme combinations. This previous work relied on the use of plasmids to express the heterologous genes in *S. cerevisiae* BY4741. However, the use of plasmids is less desirable for metabolic engineering purposes due to their instability and constant selective pressure for maintenance. Hereupon, the same pathway was integrated using CRISPR-Cas9 into *S. cerevisiae* IMX581, a strain with integrated Cas9.^30^

This new strain (JI1 strain) produced 762.0 ± 59.2 μg/L of curcumin in Yeast Nitrogen Base (YNB) minimal medium when supplemented with 30 mg/L ferulic acid (Table 1). The higher curcumin production obtained previously using plasmids (2.7 mg/L^17^) is likely attributed to the high copy number of plasmids inside the cell, resulting in a high genetic expression of curcumin biosynthetic genes and, consequently, a higher curcumin yield. Next, we introduced the COMT enzyme allowing the curcumin biosynthesis from caffeic acid (JI2 strain). During the development of this study, only *A. thaliana* COMT (*At*COMT) has been expressed in yeast to construct a biosynthetic pathway for synthesizing vanillin.^31^ Consequently, we chose the same enzyme for the construction of the curcumin artificial biosynthetic pathway. Meanwhile, other COMT enzymes were expressed in yeast including the ones from *Nicotiana tabacum* and *Rehmannia glutinosa*.^29,32^ Supplementation of 30 mg/L caffeic acid to JI2 strain resulted in the production of 497.6 ± 35.7 μg/L of curcumin. The absence of any other curcuminoids indicates that the *Pp*FerA, *Cl*DCS, and *Cl*CURS1 enzymes cannot utilize caffeic acid to produce alternative curcuminoid compounds. Subsequently, to synthesize curcumin from *p*-coumaric acid, 4-hydroxyphenylacetate 3-monooxygenase reductase component (HpaC) from *Salmonella enterica* (*Se*HpaC) and the native version of 4-hydroxyphenylacetate 3-monooxygenase oxygenase component (HpaB) from *Pseudomonas aeruginosa* (*Pa*HpaB) which have previously demonstrated high proficiency in caffeic acid synthesis in yeast,^33^ were selected to construct the JI3 strain. The supplementation of 30 mg/L *p*-coumaric acid resulted in 88.4 ± 26.0 μg/L of curcumin, a significantly lower yield compared to the yields obtained with the previous strains. When a second coumaric acid pulse at 48 h was fed, the curcumin titer increased to 128 ± 36.7 μg/L. In addition, demethoxycurcumin, a curcuminoid formed by conjugation of *p*-coumaric acid and ferulic acid derivatives, was also synthesized (14.9 ± 1.6 μg/L) revealing the capacity of the enzymes *Pp*FerA, *Cl*DCS and *Cl*CURS to use *p*-coumaric acid to synthesize other curcuminoids, although with significantly lower efficiency (Table 1). In conclusion, we successfully validated, for the first time, the biosynthesis of curcumin in an engineered yeast through the supplementation of the phenylpropanoids caffeic acid and *p*-coumaric acid.

**Table 1:** Curcumin Production by Different *S. cerevisiae* Strains after 72 h^a^

strain	substrate	[curcuminoid] (μg/L)
JI1: IMX581; + *Pp*FerA; + *Cl*DCS; + *Cl*CURS1	ferulic acid (30 mg/L)	[Cur] = 762.0 ± 59.2
JI2: JI1; + *At*COMT	caffeic acid (30 mg/L)	[Cur] = 497.6 ± 35.7
JI3: JI2; + *Pa*HpaB; + *Se*HpaC	*p*-coumaric acid (30 mg/L)	[Cur] = 88.4 ± 26.0
	*p*-coumaric acid (30 + 30 mg/L)	[Cur] = 128.0 ± 36.7
		[DMC] = 14.9 ± 1.6

a Cur: curcumin, DMC: demethoxycurcumin, PpFerA: feruloyl-CoA synthetase from Pseudomonas paucimobillis; ClDCS: diketide-CoA synthase from *C. longa*, ClCURS1: curcumin synthase 1 from *C. longa*, AtCOMT: caffeic acid O-methyltransferase from *Arabidopsis thaliana*; PaHpaB: 4-hydroxyphenylacetate 3-monooxygenase oxygenase subunit B from *P. aeruginosa*, SeHpaC: 4-hydroxyphenylacetate 3-monooxygenase reductase subunit C from *S. enterica*. The presented average values ± standard deviations were derived from three independent biological replicates.

**Figure 2:**
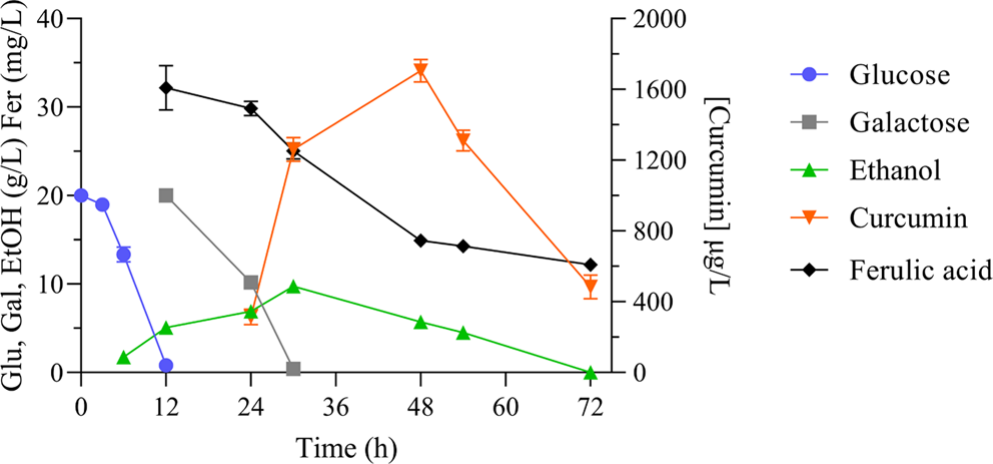
Metabolic profile and curcumin production of strain JG1 cultivated in YPD medium with 20 g/L glucose. At 12 h, 2% galactose and 30 mg/L ferulic acid were added to the media. Glu: Glucose, Gal: Galactose, EtOH: Ethanol, Fer: Ferulic acid. The presented average values ± standard deviations were derived from three independent biological replicates.

In parallel, to examine an alternative promoter control for curcumin biosynthesis in *S. cerevisiae*, the genes *Pp*FerA, *Cl*DCS and *Cl*CURS1 were placed under control of galactose inducible promoters (GAL 1,10) and were integrated into the IMX581 genome, resulting in strain JG1. The induction of curcumin biosynthesis in JG1 strain was initiated by adding 20 g/L galactose and 30 mg/L of ferulic acid at 12 h. However, contrarily to what was observed with JI strains using constitutive promoters, as JG1 fermentation progressed, the intensity of the yellowish color, indicative of curcumin presence, gradually decreased between 48 and 72 h, prompting us to monitor curcumin production over time. Indeed, curcumin was first detected at 24 h and peaked at 48 h, reaching 1705.4 ± 63.6 μg/L, after which it began declining, reaching a concentration of 484.8 ± 68.1 μg/L at 72 h (Figure 2). Galactose inducible promoters are activated by glucose depletion in the presence of galactose. Complete galactose depletion was observed at 48 h, which means that the expression of genes from curcumin biosynthetic pathway was inactivated from this point. Nevertheless, the inactivation of the expression itself does not justify the curcumin degradation. Hereupon, we hypothesize that the curcumin degradation might be caused by autoxidation^34^ or by any yeast endogenous enzyme. Regarding the second hypothesis, while *E. coli* features the *curA* gene encoding curcumin reductase (CurA), there is no reported gene responsible for curcumin degradation identified in *S. cerevisiae*. Upon subjecting CurA sequence (NCBI Reference Sequence: NP_415966.6) to a protein BLAST against the *S. cerevisiae* S288C proteome, the outcome revealed a low match with an uncharacterized protein (*E*-value = 9e^–22^, Identity = 25%, Positives = 43% and cover = 97%) with the systematic name YML131W and with possible oxidoreductase activity. The alignment is presented in Supporting File 2. These findings suggest a possible role of YML131W linked to curcumin degradation in *S. cerevisiae*. However, further studies are required to clearly understand the function of YML131W. Nevertheless, the utilization of the GAL system led to higher curcumin production at its peak compared with the constitutive control strain (JI1). Further investigation using this system, particularly through the deletion of gal80 repressor to eliminate the dependency on galactose induction,^35^ is necessary to comprehensively assess its potential for enhancing curcumin production. Hereupon, we decided to further investigate curcumin biosynthesis by expressing genes under the control of constitutive promoters since no curcumin degradation was observed, thus suggesting that in these strains, the rate of curcumin degradation is slower than the rate of its production.

### 2.2 *De Novo* Curcumin Biosynthesis

**Figure 3:**
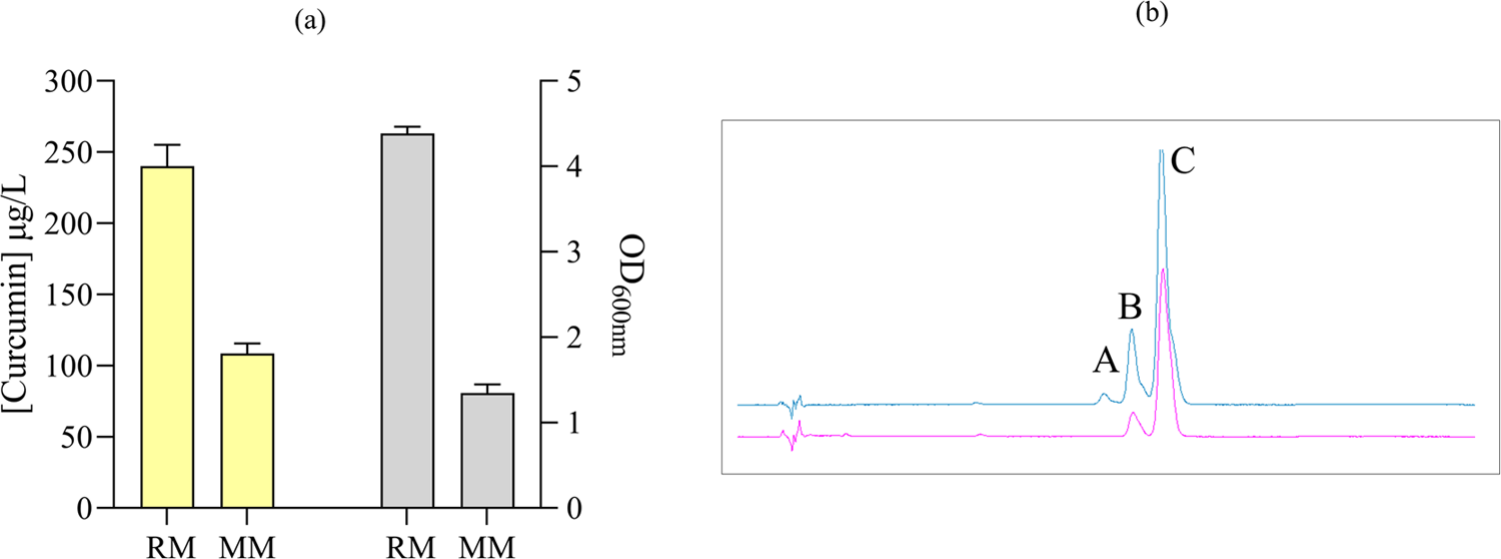
(a) Curcumin production (yellow bars) and optical density at 600 nm (OD_600 nm_) (gray bars) obtained after 72 h fermentation by JQ1 strain in rich (RM) and minimal (MM) media. The presented average values ± standard deviations were derived from three independent biological replicates. (b) HPLC analysis of the curcuminoid standard (blue spectrum) and curcuminoid extract from JQ1 (purple spectrum). Identification of peaks stands for A: bisdemethoxycurcumin, B: demethoxycurcumin, and C: curcumin.

After the verification of curcumin biosynthesis resulting from the supplementation of phenylpropanoids, we evaluated if *S. cerevisiae* was capable of producing curcumin from glucose. To accomplish this, an engineered *p*-coumaric acid overproducing strain (QL158) was strategically employed.^28^ This strain was genetically engineered for *de novo* production of high amounts of *p*-coumaric acid through the optimization of the metabolic flux toward the biosynthesis of the aromatic amino acids, tyrosine, and phenylalanine, and by the introduction of TAL from *Flavobacterium johnsoniae* and PAL and C4H from *A. thaliana*. In our preliminary assays, the QL158 strain produced 310.6 ± 18.4 mg/L of *p*-coumaric acid (rich media, yeast extract Peptone Dextrose (YPD)) and 213.2 ± 18.4 mg/L (minimal media – YNB). Hereupon, we introduced the previously studied genes (*Pa*HpaB, *Se*HpaC, *At*COMT, *Pp*FerA, *Cl*DCS, and *Cl*CURS1) in the QL158 strain, thus creating the JQ1 strain (Figure 1). The *de novo* curcumin production was evaluated in minimal YNB and in rich YPD media. In minimal media, curcumin production reached 108.5 ± 7.0 μg/L, while in rich media, the production reached 240.1 ± 15.1 μg/L (Figure 3a). Moreover, demethoxycurcumin was also detected in extracts, reaching 13.5 ± 0.4 μg/L in minimal media and 22.0 ± 0.3 μg/L in rich media (Figure 2b). No degradation of curcuminoids was observed over time. Relatively to the synthesis of phenylpropanoids (Table S1), at 24 h, *p*-coumaric acid significantly accumulated, reaching 141.5 ± 38.8 and 257.0 ± 14.2 mg/L, in minimal and rich media, respectively. At the end of fermentation, *p*-coumaric concentrations attained 125.0 ± 27.4 mg/L (minimal media) and 146.7 ± 17.1 mg/L (rich media). Caffeic acid concentrations were significantly lower than *p*-coumaric acid concentrations; however, a slight increase during the fermentation course was observed, accumulating at 8.1 ± 1.8 and 11.5 ± 1.4 mg/L in minimal and rich media, respectively. The last phenylpropanoid, ferulic acid, was not detected in minimal media, and in rich media, it was only detected after 48 h in very low concentrations. Overall, *de novo* curcumin biosynthesis was achieved in both minimal and rich media. The highest curcumin levels were obtained in rich media possibly due to the highest cell concentration as previously stated.^17^ The substantial accumulation of *p*-coumaric acid suggests that its hydroxylation into caffeic acid was inefficient, as previously observed, potentially hindering the biosynthesis of curcumin. From this point forward, all fermentations were conducted using YPD-rich media, as it is a cheaper culture media and it allowed production of the highest curcumin titers.

### 2.3 Enhancing *p*-Coumaric Acid Conversion to Caffeic Acid

**Figure 4:**
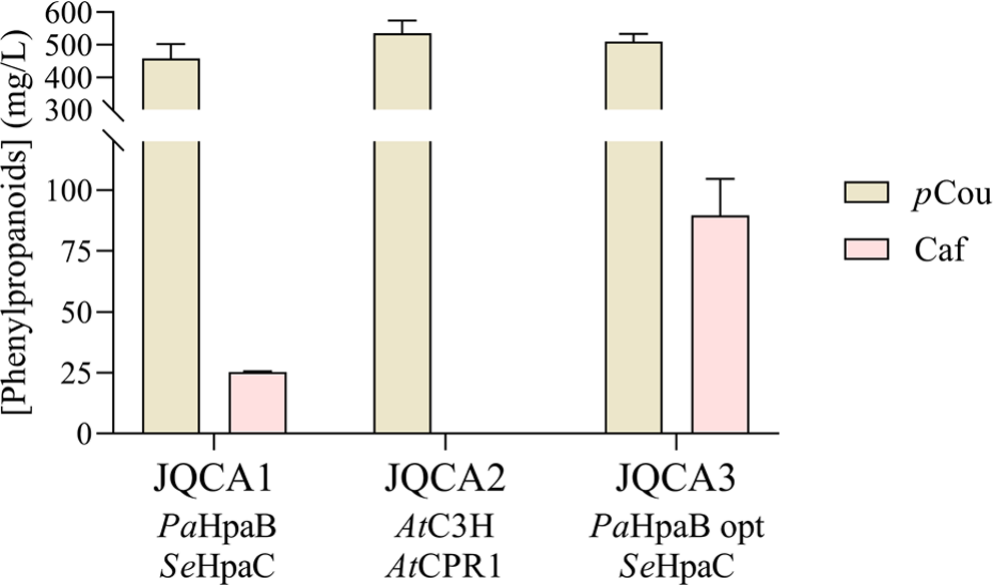
(a): Caffeic acid and *p*-coumaric production by strains JQCA1, JQCA2, and JQCA3 were cultivated in rich media with 20 g/L glucose after 72 h of fermentation. The presented average values ± standard deviations were derived from three independent biological replicates. *p*Cou: *p*-Coumaric acid, Caf: Caffeic acid.

Since the conversion of *p*-coumaric acid into caffeic acid was not efficient in strain JQ1 and in strain JI3 and the curcumin biosynthesis dropped significantly when *p*-coumaric acid was used as a substrate, we explored the possibility of enhancing this reaction by using a different enzyme. For that purpose, the caffeic acid biosynthesis was compared in QL158 expressing native versions of *Pa*HpaB and *Se*HpaC (JQCA1) (as in the strain JQ1 when the complete pathway was integrated), with QL158 expressing C3H and cytochrome P450 reductase (CPR1) (JQCA2) from *A. thaliana*.^36^ However, while the JQCA1 strain produced 25.3 ± 0.26 mg/L caffeic acid, no caffeic acid was detected in JQCA2, regardless of a high accumulation of *p*-coumaric acid. This lack of production may be attributed to the incorrect expression of C3H in *S. cerevisiae*, which is a plant-specific cytochrome P450-dependent enzyme, despite the codon-optimization.^36^ As attempts to synthesize caffeic acid through C3H and CPR proved unsuccessful, we opted to revert to our initial approach. Consequently, we decided to assess the impact of codon optimization on the *Pa*HpaB gene as stated by Zhou et al.^37^ In fact, the replacement of *Pa*HpaB with its codon-optimized version (*Pa*HpaB (opt)) (JQCA3) significantly increased the production of caffeic acid to 89.7 ± 15.1 mg/L, nearly tripling the titer (Figure 4). Moreover, Chen et al. achieved comparable levels of caffeic acid by expressing the same enzymes in a *p*-coumaric acid overproducing strain.^29^

**Figure 5:**
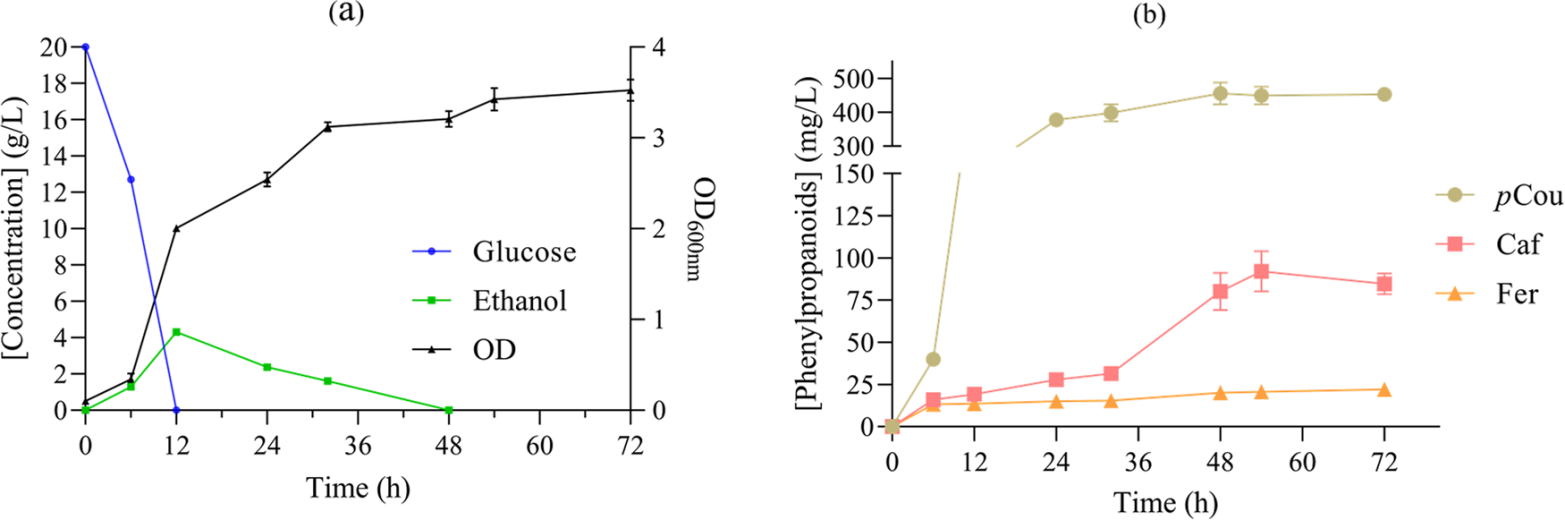
(a) Metabolic profile and optical density at 600 nm (OD_600 nm_) of strain JQ2 in rich media with 20 g/L glucose and (b) production of phenylpropanoids during fermentation. The presented average values ± standard deviations were derived from three independent biological replicates. *p*Cou: *p*-Coumaric acid, Caf: Caffeic acid and Fer: Ferulic acid.

After all, to accomplish *de novo* biosynthesis of curcumin, we replaced *Pa*HpaB by its codon-optimized version in JQ1 creating JQ2. Fermentation of JQ2 in YPD-rich medium resulted in the synthesis of 861.9 ± 113.4 μg/L curcumin from 20 g/L glucose. Additionally, 44.3 ± 4.3 μg/L of demethoxycurcumin were obtained. The replacement of *Pa*HpaB with its codon-optimized version led to a 3-fold increase in curcumin production. Evaluating the JQ2 strain metabolic profile, glucose depletion was observed at 12 h, simultaneous with the ethanol peak of 4.2 ± 0.08 g/L, and complete exhaustion of ethanol was achieved at 48 h (Figure 5a). Notably, the manifestation of curcumin production became visible around the 36th hour, evident through the development of a yellowish color in the culture medium. The detection of curcumin coincided with the phase of ethanol consumption, indicating that its production may occur during this phase such as the production of other polyphenols in *S. cerevisiae*.^38^ Throughout the fermentation, the accumulation of *p*-coumaric acid was observed during the exponential growth phase, attaining a concentration of 377 ± 20.1 mg/L at 24 h. However, the concentrations of both caffeic acid and ferulic acid remained relatively low, registering 27.9 ± 1.6 and 15.1 ± 0.18 mg/L, respectively. During the stationary growth phase and ethanol consumption phase, there was an increment in the levels of caffeic acid, culminating in a maximal concentration of 85.3 ± 11.9 mg/L at 54 h. By the end of the fermentation, a high accumulation of *p*-coumaric acid was evident, reaching a substantial concentration of 452 ± 10.3 mg/L (Figure 5b).

**Figure 6:**
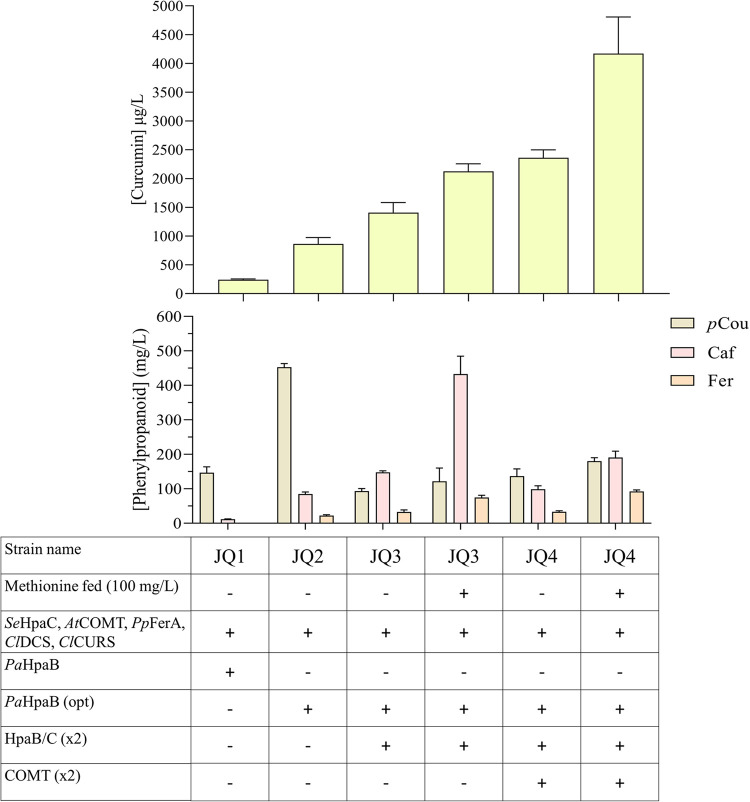
*De novo* curcumin production and phenylpropanoid accumulation of JQ1, JQ2, JQ3, and JQ4 in rich media with 20 g/L of glucose after 72 h of fermentation. *Se*HpaC: 4-hydroxyphenylacetate 3-monooxygenase reductase component from *S. enterica*; *At*COMT: Caffeic acid *O*-methyltransferase from *A. thaliana*; *Pp*FerA: feruloyl-CoA synthetase from *P. paucimobilis*; *Cl*DCS: diketide-CoA synthase, *Cl*CURS1: curcumin synthase 1 from *C. longa*; *Pa*HpaB: 4-hydroxyphenylacetate 3-monooxygenase oxygenase component; *Pa*HpaB (opt): *Pa*HpaB codon-optimized; *p*Cou: *p*-coumaric acid; Caf: Caffeic acid; Fer: Ferulic acid. The presented average values ± standard deviations were derived from three independent biological replicates.

In response to the persistent substantial accumulation of *p*-coumaric acid by JQ2 and to increase the fluxes through the curcumin biosynthetic pathway, an additional copy of *Pa*HpaB (opt) and *Se*HpaC genes was introduced in JQ2. This culminated in the development of a derivative strain denoted as JQ3. The supplementary genetic copy instigated the synthesis of curcumin, yielding a titer of 1.4 ± 0.17 mg/L (1408.6 μg/L), thus representing an enhancement of 77% compared to the previous strain JQ2. Demethoxycurcumin levels also increased reaching 133.8 ± 4.9 μg/L. Furthermore, colonies of JQ3 growth on solid media during plasmid curing exhibited a yellow color, a characteristic not observed in the preceding strains (Figure S1). Remarkably, the augmented HpaB/C genetic charge resulted in a considerable reduction in the accumulation of *p*-coumaric acid to 93.4 ± 7.4 mg/L (Figure 6), indicative of an increased conversion to caffeic acid due to a higher hydroxylation activity. Accumulation of caffeic acid reached 147.9 ± 4.4 mg/L, 1.7 times higher than that of JQ2. As observed for JQ2, at 24 h, during exponential growth, *p*-coumaric acid was the main phenylpropanoid. After this phase, there was a subsequent increase in caffeic acid concentrations, doubling from the 24 h time point and the end of fermentation (Table S1). Additionally, the accumulation of ferulic acid at 72 h increased 1.5 times to 32.7 ± 5.9 mg/L, relative to JQ2 (Figure 6). Concluding, the extra copy of HpaB/C improved the fluxes through the curcumin biosynthetic pathway increasing the biosynthesis of caffeic and ferulic acid and consequently curcumin titers.

### 2.4 Methylation of Caffeic Acid to Ferulic Acid

The conversion of caffeic acid to ferulic acid involves a crucial methylation step, wherein a methyl group is transferred to a ferulic acid molecule. In yeast, the methyl group donor for methylation reactions is linked to the *S*-adenosylmethionine (SAM) cycle. SAM serves as the primary methyl group donor in various cellular methylation reactions. As the conversion of caffeic acid to ferulic acid requires a methyl group transfer, the availability of SAM becomes critical in determining the efficiency of this reaction. To enhance the SAM pool and thereby boost the conversion of caffeic acid to ferulic acid, the supplementation of methionine, the precursor of SAM, has emerged as a strategy. In fact, the supplementation of 100 mg/L of methionine to JQ3 increased curcumin yield to 2.2 ± 0.13 mg/L (2125.7 μg/L), representing a 1.6 improvement in production (Figure 6). In addition, ferulic acid accumulation increased to 75.0 ± 6.2 mg/L (2.3-fold higher than without methionine supplementation), revealing that methionine elevated the methylation efficiency of *At*COMT. Interestingly, caffeic acid levels reached 432.7 ± 51.9 mg/L, almost 3 times more than without methionine supplementation (Figure 6). This might be attributed to an increased flow through the *At*COMT reaction, which alleviated the bottleneck in the previous step, leading to elevated levels of caffeic acid. Therefore, the final concentrations of caffeic and ferulic acid significantly increased (Figure 6). Moreover, when methionine was fed, significantly less demethoxycurcumin was produced, with only trace amounts detected. This could mean that when ferulic acid availability surpasses a certain threshold, *p*-coumaric acid may not be used for curcuminoid synthesis.

### 2.5 Precursor Supply Enhancement

The availability of the extender molecule malonyl-CoA is essential for curcumin biosynthesis. To assess whether the availability of malonyl-CoA was restricting curcumin production, we overexpressed the native genes of cytosolic acetyl-CoA carboxylase (ACC1) and fatty-acyl coenzyme A oxidase (FOX1) within the JQ3 strain by using a plasmid system. ACC1 is responsible for the direct conversion of acetyl-CoA to malonyl-CoA,^39^ and FOX1 is a key enzyme in fatty acid β-oxidation. FOX1 may increase malonyl-CoA by increasing levels of precursor acetyl-CoA, a strategy already employed to promote carminic acid biosynthesis that requires seven malonyl-CoA molecules in yeast.^40^ Plasmids harboring ACC1 and FOX1 alone or together were transformed into the JQ3 strain, and the curcumin biosynthesis was evaluated in minimal media (YNB), required for plasmid maintenance, and compared with JQ3 harboring the empty plasmid (pSP-GM1). However, either overexpression of ACC1 or FOX1 or both together did not increase curcumin production relative to the control (Figure S2). Overexpressing native ACC1 gene was found to enhance the levels of malonyl-CoA-dependent molecules in yeast.^39,41^ Nevertheless, studies have highlighted the use of a feedback-resistant ACC1 mutant version to further augment the malonyl-CoA pool.^42^ The phosphorylation of ACC1 by Snf1 reduces its activity, and Snf1 is activated in response to glucose depletion.^43^ Since curcumin synthesis occurs during the ethanol phase, exploring the use of mutated ACC1 as a potential strategy for improvement of curcumin yields is worth considering in the future. In addition, despite the efforts to enhance malonyl-CoA availability through overexpressing ACC1 and FOX1 genes, the lack of significant increase in curcumin production could also suggest that, at this stage, malonyl-CoA availability might not be the limiting factor for curcumin biosynthesis.

### 2.6 Increasing Flux through Ferulic Acid Biosynthesis

Since in JQ3, caffeic acid was the highest accumulated phenylpropanoid, we tested the same strategy as previously by introducing an extra copy of *At*COMT to increase caffeic acid conversion to ferulic acid. The resulting strain was labeled as JQ4. As previously, the extra copy *At*COMT elevated curcumin levels to 2.4 ± 0.1 mg/L (2360.7 μg/L), 1.7 times more than in JQ3 (Figure 6). Again, only traces of demethoxycurcumin were detected. Furthermore, less caffeic acid was accumulated relatively to JQ3, reaching 98.7 ± 10.1 mg/L, hence revealing a higher capacity for ferulic acid biosynthesis. Nevertheless, ferulic acid accumulation levels were similar in JQ3 and JQ4, indicating that the extra ferulic acid produced was utilized for the synthesis of curcumin (Figure 6). Methionine supplementation to JQ4 elevated the levels of curcumin 1.8 times to 4.2 ± 0.6 mg/L (4171.3 μg/L). Moreover, a higher accumulation of all phenylpropanoids was detected (Figure 6). Notably, ferulic acid accumulation reached 92.2 ± 4.3 mg/L (2.8 times more than that without methionine supplementation) (Figure 6). Overall, besides increased expression of *At*COMT, the supplementation of methionine increased the levels of ferulic acid. This suggests that SAM availability still might have been a limiting factor for ferulic acid synthesis. Chen et al.^29^ demonstrated that the augmentation of COMT activity could be obtained by integrating more copies of the gene. Specifically, the integration of four copies led to increased ferulic acid yields in their study. Here, it was demonstrated that enhancing the expression levels of phenylpropanoid pathway genes improves the fluxes through curcumin biosynthesis. Nevertheless, a significant accumulation of phenylpropanoid precursors was observed. Chen et al.^29^ also showed that enhancing the supply of cofactors essential for the phenylpropanoid pathway–namely NADH, FADH2, and SAM- resulted in a substantial increase in ferulic acid biosynthesis. In the future, a more deliberate and systematic approach should be explored to effectively direct the flux toward curcumin synthesis either by defining the optimal expression levels for each pathway gene (fine-tuning) or by using a different promoter system. Moreover, strategies to enhance the bioavailability of redox cofactors, SAM, and the extended substrate malonyl-CoA must be considered. Overall, we developed a *S. cerevisiae* strain capable of producing curcumin without requiring the supplementation of phenylpropanoids precursors. Our final strain (JQ4) produced 2.4 ± 0.1 mg/L curcumin from 20 g/L of glucose. Methionine supplementation to JQ4 elevated curcumin yield to 4.2 mg/L. Prior to our study, *de novo* curcumin biosynthesis had only been achieved in genetically modified *E. coli*.^12,21^

In recent years, curcumin has emerged as a promising natural therapeutic agent because of its diverse biological effects. Using microbes to produce curcumin offers a solution to the challenges linked with curcumin plant extraction. Microbial production might enable a rapid, cost-effective, and eco-friendly way to produce curcumin mainly when simple substrates are used. In our earlier research, we assessed the production of curcumin from supplemented ferulic acid, identifying the most efficient enzymes for the process. Nonetheless, the dependency on plasmids for curcumin synthesis poses limitations. Therefore, to overcome such limitations, herein, we integrated the curcumin genes into the yeast genome and further confirmed the synthesis of curcumin from ferulic acid. Next, we introduced the genes responsible for the curcumin biosynthesis from the upstream intermediates, namely *p*-coumaric and caffeic acids. After the curcumin biosynthesis was successfully confirmed, this same pathway was integrated into a *p*-coumaric acid overproducing strain. To overcome the accumulation of intermediate phenylpropanoids, the expression of HpaB/C and *At*COMT was increased by expressing an extra copy of each gene. Also, the expression of curcumin biosynthetic genes under the control of galactose inducible promoters revealed the possible existence of an endogenous yeast gene capable of degrading curcumin (YML131W). In the future, the deletion of this gene could provide important insights into its role in curcumin degradation. In conclusion, we report for the first time the *de novo* biosynthesis of curcumin in *S. cerevisiae* achieving up to 4.2 mg/L. By integrating genes from different organisms into the *S. cerevisiae* genome, we constructed an artificial pathway for *de novo* curcumin biosynthesis from glucose. Indeed, the *de novo* production of curcumin holds the potential for significant further improvement by additional genetic modifications, such as the fine-tuning of curcumin pathway genes. The fine-tuning may be performed by multiple integrations of target genes, for instance, via library construction by multiple integration at delta sites using CRISPR-Cas9. This approach generates a mutant library containing various copies of the target gene. After library construction, the best producer must be evaluated by qPCR to determine the optimal number of copies of the targeted gene. This strategy can be iteratively applied to each gene within the pathway, spanning from HpaB/C to *Cl*CURS. Additionally, the titer of curcumin can be substantially increased by scaling up the fermentation process. Finally, we believe that our work represents a good basis for the development of an extremely efficient cell factory for microbial curcumin production.

## 3 Materials and Methods

### 3.1 Genes, Plasmids, and Strains

The native yeast genes encoding ACC1 and FOX1 were PCR-amplified from *S. cerevisiae* genomic DNA. The genes *Se*HpaC and *Pa*HpaB were amplified by colony PCR from strains available in our lab. Genes *Cl*DCS and *Cl*CURS and *Pp*FerA were previously codon-optimized for *S. cerevisiae*.^17^*At*COMT and *Pa*HpaB (opt) were also codon-optimized for *S. cerevisiae* and PCR-amplified from synthetic fragments (TWIST Bioscience). All gene sequences are presented in Table S2. Genes were cloned using restriction ligation or by Gibson assembly and confirmed via colony PCR, digestion, and sequencing.

The plasmids pSP-GM1 (accession number: Addgene #64739)^44^ and pBEVY_GL (accession number: Addgene #51225)^45^ were used for the construction of the integration cassettes, placing the genes under the control of the respective promoters. The plasmid pSP-GM1 was also used for the overexpression of *S. cerevisiae* native genes. Guide RNA (gRNA) plasmids were kindly provided by Liu et al.^28^ targeting genomic loci where heterologous genes could be efficiently and stably expressed.

*E. coli* NZY5α (NZYTech, Portugal) was used for plasmid cloning, storage, and propagation. *S. cerevisiae* IMX581^30^ and QL158^28^ (both with Cas9 integrated) were used as host strains for the integration of curcumin biosynthetic pathways. Plasmids, *S. cerevisiae* strains, and primers sequences used in this work are listed in Tables S3, S4, and S5, respectively.

### 3.2 Strain Construction

For strain construction, genomic integration was mediated by CRISPR-Cas9 technology.^30^ Integration cassettes harboring the (promoter-gene-terminator)*_n_* were amplified from the corresponding constructed plasmid with a forward primer containing 25-bp homology to the upstream homology arm and a reverse primer with 25-bp homology to the downstream homology arm of the specific integration site. For instance, for the construction of JQ01, a cassette harboring p_TDH3_-*Cl*CURS1-t_synt27_-t_ADH1_-*Cl*DCS-p_PGK1_-p_TEF_-PpFerA-t_CYC_ was amplified from pSP-GM1_FerA_DCS_CURS using XII-4_pTDH3_FW and XII-4_tCYC_RV primers. Parallelly, the specific upstream and downstream homologous regions were amplified from *S. cerevisiae* genomic DNA, for this case using XII-4_US_FW/RV and XII-4_DS_FW/RV. Next, the three fragments (100 ng/kb) were cotransformed in QL158 together with 1000 ng of the specific gRNA plasmid (pQLC010, in this case) using lithium acetate method^46^ for *in vivo* assembly and simultaneous gap repair. The resulting strains were selected in YNB minimal media (20 g/L glucose, 6.7 g/L YNB, and 0.10 g/L uracil) agar plates (15 g/L), and the pathway integration confirmed via PCR of extracted genomic DNA. After confirmation, gRNA plasmids were cured by plating with 1 g/L 5-fluoroorotic acid (5-FOA).

### 3.3 Strain Cultivation and Curcumin Extraction

*E. coli* was cultivated at 37 °C at 200 rpm in Lysogeny Broth (LB) Lennox containing 100 μg/mL of ampicillin. *S. cerevisiae* was cultivated in a YPD medium containing 20 g/L peptone, 10 g/L yeast extract, 20 g/L glucose, or YNB minimal media containing the same concentration of glucose. For shake-flask fermentation, a 5 mL preculture was grown overnight at 30 °C, 200 rpm and used to inoculate 250 mL flasks containing 50 mL of media (YPD or YNB) with an initial Optical Density at 600 nm (OD_600_) of 0.1. For galactose induction, 2% galactose was supplemented at 12 h. When necessary, ferulic acid, caffeic acid, or *p*-coumaric acid were supplemented at 24 h at a final concentration of 30 mg/L. Shake-flask fermentations were maintained for 72 h. Samples were centrifuged, and the supernatant was used to quantify phenylpropanoids, sugars, and ethanol. Curcumin was extracted from cell pellets at the final time point, unless otherwise specified, using methanol as previously described.^17^

### 3.4 Metabolite Analysis

For quantification of curcuminoids, *p*-coumaric acid, caffeic acid, and ferulic acid, UHPLC was performed as previously reported.^17^ The method used for ferulic acid quantification was employed for the other phenylpropanoids. Caffeic acid, *p*-coumaric acid, and ferulic acid were detected at 310 nm with retention times of 6.0, 8.0, and 8.7 min, respectively. Curcuminoids were detected at 425 nm with detection times of 11.8 min for bisdemethoxycurcumin, 12.7 min for demethoxycurcumin, and 13.5 min for curcumin. HPLC was used to quantify glucose, galactose, and ethanol. The chromatographic system was composed by Shimadzu LC-2060C equipped with Biorad Aminex HPX-87H (300 mm × 7.8 mm, 9 μm particle size) column. The oven temperature was set to 60 °C. The mobile phase was composed of 5 mM of sulfuric acid at flow rate of 0.6 mL/min for 25 min. Glucose was detected at 8.8 min, galactose at 9.5 min, and ethanol at 21.8 min.

### 3.5 Bioinformatic Analysis

Basic Local Alignment Search Tool (BLAST) analysis was conducted using protein–protein BLAST (blastp) algorithm available in the NCBI BLAST tool (https://blast.ncbi.nlm.nih.gov). The *E. coli* CurA protein sequence (NCBI Reference Sequence: NP_415966.6) was compared with the reference proteome of *S. cerevisiae* S288C found in the nonredundant protein sequences (nr) database. The algorithm parameters were set to default values with the exception of word size, which was specifically set to 3.

## Supporting Information Available

Phenylpropanoids produced during fermentation by engineered *S. cerevisiae* strains for *de novo* curcumin production; sequence of the genes used; plasmids used; strain JQ3 growth in solid minimal media exhibiting yellow biomass due to curcumin production and a biomass pellet of JQ3 after fermentation in rich media; curcumin produced by strain JQ3 expressing plasmids harbouring different genes (PDF)
*E. coli* CurA protein BLAST result against the *S. cerevisiae* S288C proteome (TXT)
