# Supplementary material for: De Novo Biosynthesis of Curcumin in Saccharomyces cerevisiae
Source: ACS Synth Biol. 2024 May 24;13(6):1727–36. doi: 10.1021/acssynbio.4c00059 (PMC11197086; doi:10.1021/acssynbio.4c00059)
Supplement: Supplementary file 1 — sb4c00059_si_001.pdf [file sb4c00059_si_001.pdf]

Supplementary Material

De novo biosynthesis of curcumin in *Saccharomyces cerevisiae*

João Rainha[1], Joana Rodrigues[1,2*] and Lígia Rodrigues[1,2]

[1] Centre of Biological Engineering, University of Minho, Braga 4710-057, Portugal
[2] LABBELS – Associate Laboratory, Braga, Portugal

*Corresponding author:
Dr. Joana L. Rodrigues - *CEB - Centre of Biological Engineering, University of Minho, 4710-057 Braga, Portugal.*
ORCID: 0000-0002-3217-2320
Email: joanarodrigues@deb.uminho.pt

Supplementary Files

Supplementary File 1: Supplementary figures and tables.

Supplementary File 2: *Escherichia coli* CurA protein BLAST result against the *S. cerevisiae* S288C proteome.

Table S1: Phenylpropanoids produced during fermentation by engineered *Saccharomyces cerevisiae* strains *for de novo* curcumin production. The presented average values ± standard deviations were derived from three independent biological replicates.

			[Phenylpropanoid] (mg/L)		
Strain	Media	Time point	*p*-Coumaric acid	Caffeic acid	Ferulic acid
JQ1	Rich media	24 h	257.0 ± 14.2	6.3 ± 0.31	-
		48 h	218.0 ± 19.1	9.9 ± 1.1	1.9 ± 0.2
		72 h	146.7 ± 17.1	11.5 ± 1.4	1.7 ± 0.2
JQ1	Minimal media	24 h	141.5 ± 38.8	5.7 ± 1.9	-
		48 h	150.8 ± 48.0	6.5 ± 2.3	-
		72 h	125.0 ± 27.4	8.1 ± 1.8	-
JQ2	Rich media	24 h	377.5 ± 20.1	27.9 ± 1.6	15.1 ± 0.18
		48 h	455.8 ± 32.9	80.2 ± 11.0	20.1 ± 1.0
		72 h	452.9 ± 10.3	84.7 ± 6.3	22.0 ± 2.7
JQ3	Rich media	24 h	169.4 ± 3.0	62.5 ± 0.9	23.4 ± 0.3
		48 h	57.5 ± 3.1	101.8 ± 6.7	20.1 ± 0.7
		72 h	93.4 ± 7.4	147.9 ± 4.4	32.7 ± 5.9
JQ3	Rich media + 100 mg/L methionine	24 h	151.9 ± 4.1	68.5 ± 5.6	26.4 ± 2.9
		48 h	168.9 ± 17.9	206.7 ± 18.5	50.7 ± 8.7
		72 h	121.5 ± 38.6	432.7 ± 51.9	75.0 ± 6.2
JQ4	Rich media	24 h	117.5 ± 5.4	48.8 ± 2.3	37.1 ± 1.4
		48 h	86.3 ± 5.7	89.4 ± 7.7	28.4 ± 1.4
		72 h	136.9 ± 20.8	98.7 ± 10.1	33.2 ± 3.1
JQ4	Rich media + 100 mg/L methionine	24 h	144.2 ± 27.5	59.2 ± 5.3	36.2 ± 5.2
		48 h	231.3 ± 35.1	140.1 ± 0.13	66.0 ± 3.8
		72 h	180.3 ± 10.1	190.9 ± 18.4	92.2 ± 4.3

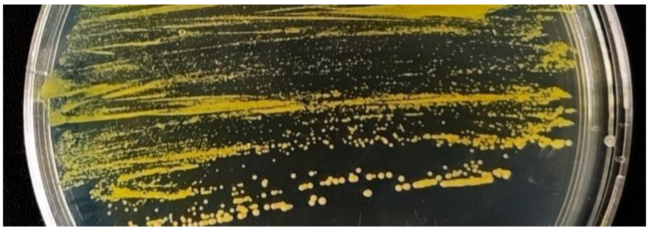

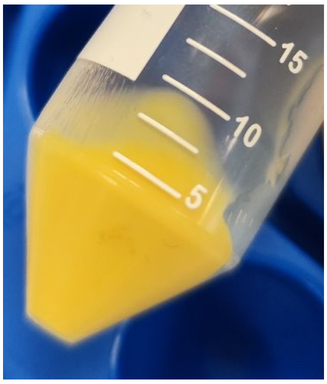

Figure S1: Strain JQ3 growth in solid minimal media exhibiting yellow biomass due to curcumin production and a biomass pellet of JQ3 after fermentation in rich media.

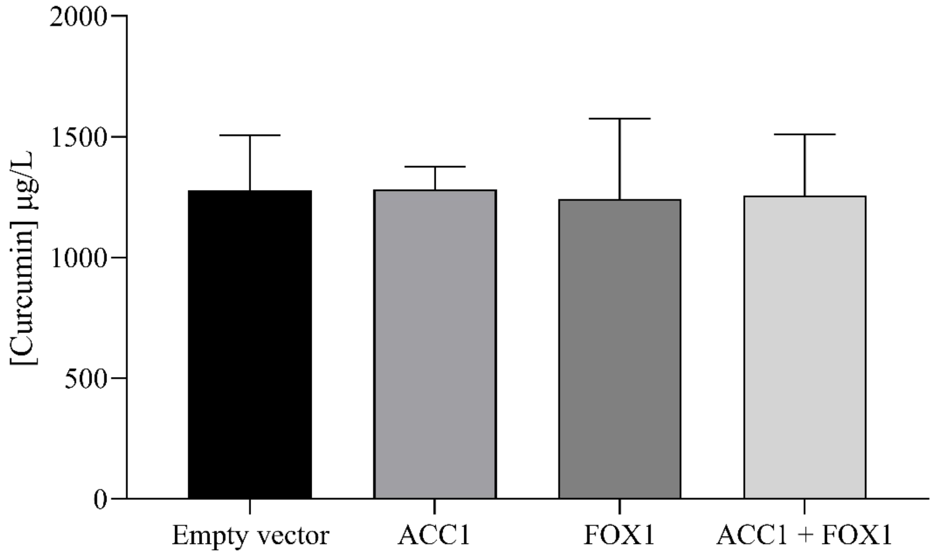

Figure S2: Curcumin produced by strain JQ3 expressing plasmids harbouring different genes. ACC1: Acetyl-CoA carboxylase; FOX1: Fatty-acyl coenzyme A oxidase; MET13: methylenetetrahydrofolate reductase. The presented average values ± standard deviations were derived from three independent biological replicates.

Table S2: Sequence of the genes used in this work. “Opt" Represents Codon-optimized genes.

1- Gene name<br>2- Organism	Sequence 5’-3’
1- *Pa*HpaB<br>2- *Pseudomonas aeruginosa*	ATGAAACCCGAAGATTTCCGTGCCTCTGCCACCCGTCCGTTCACCGGCGAGGAGTACCT<br>CGCCAGCCTGCGCGACGACCGTGAGATCTACATCTACGGCGACCGCGTCAAGGACGTC<br>ACCAGCCACCCCGCCTTCCGCAACGCGGCCGCCTCCATGGCCCGGCTCTACGACGCCCT<br>GCACGATCCGCAGAGCAAGGAAAAGCTCTGCTGGGAGACCGATACCGGCAACGGCGG<br>CTATACCCACAAGTTCTTCCGCTACGCGCGCAGCGCCGACGAACTGCGCCAGCAGCGC<br>GACGCCATCGCCGAGTGGTCGCGGCTGACCTACGGCTGGATGGGCCGCACCCCGGACT<br>ACAAGGCCGCCTTCGGCAGCGCCCTCGGCGCCAACCCGGGCTTCTACGGGCGTTTCGA<br>GGACAACGCGAAAACCTGGTACAAGCGCATCCAGGAAGCCTGCCTGTACCTCAACCAT<br>GCCATCGTCAACCCGCCGATCGACCGCGACAAGCCGGTGGACCAGGTCAAGGACGTGT<br>TCATCTCGGTGGACGAGGAAGTCGACGGCGGCATCGTCGTCAGCGGCGCCAAGGTGGT<br>CGCCACGAATTCCGCGCTGACCCACTACAACTTCGTCGGCCAGGGTTCGGCGCAACTGC<br>TCGGCGACAACACCGACTTCGCCCTGATGTTCATCGCGCCGATGAACACCCCCGGCATG<br>AAGCTGATCTGCCGCCCCTCCTACGAACTGGTGGCGGGTATCGCCGGATCGCCGTTCGA<br>CTACCCGCTGTCCAGCCGTTTCGACGAGAACGACGCGATCCTGGTGATGGACAAGGTG<br>TTCATCCCCTGGGAGAACGTACTGATCTACCGCGACTTCGAGCGCTGCAAGCAGTGGTT<br>CCCCCAGGGTGGCTTCGGCCGGCTGTTCCCGATGCAGGGCTGCACCCGCCTGGCGGTCA<br>AGCTCGACTTCATCACCGGCGCCCTCTACAAGGCCCTGCAATGCACCGGCTCCCTGGAG<br>TTCCGCGGCGTGCAGGCGCAGGTCGGCGAAGTGGTGGCCTGGCGCAACCTGTTCTGGT<br>CGCTGACCGACGCCATGTACGGCAACGCCAGCGAATGGCACGGCGGCGCCTTCCTGCC<br>CAGCGCCGAGGCCCTGCAGGCCTACCGCGTGCTGGCGCCGCAGGCCTACCCGGAGATC<br>AAGAAGACCATCGAGCAGGTGGTCGCCAGCGGCCTGATCTACCTGCCCTCCGGCGTTC<br>GCGACCTGCACAATCCGCAACTCGACAAGTATCTCTCCACCTATTGCCGCGGCTCCGGC<br>GGCATGGGCCACCGGGAGCGGATCAAGATCCTCAAGCTGCTCTGGGACGCCATCGGCA<br>GCGAGTTCGGCGGCCGCCACGAGCTGTACGAGATCAACTACGCCGGCAGCCAGGACGA<br>GATCCGCATGCAGGCCCTGCGCCAGGCGATCGGCAGCGGGGCGATGAAGGGCATGCTC<br>GGCATGGTCGAGCAGTGCATGGGCGACTACGACGAGAACGGCTGGACCGTGCCGCACC<br>TGCACAACCCGGACGACATCAACGTGCTCGATCGCATCCGCCAATGA
1- *Pa*HpaB (opt)<br>2- *Pseudomonas aeruginosa*	ATGAAACCAGAAGACTTTAGAGCTTCAGCTACCCGTCCCTTTACTGGTGAAGAATATTT<br>AGCATCTTTAAGGGATGATAGAGAAATTTATATTTATGGAGATCGTGTGAAAGATGTA<br>ACTTCTCATCCGGCATTTCGTAATGCCGCTGCGTCGATGGCTAGGCTTTATGATGCTTTA<br>CATGACCCACAATCTAAAGAGAAATTATGTTGGGAAACGGACACTGGAAATGGTGGTT<br>ACACTCATAAATTCTTCAGATATGCAAGATCTGCGGATGAGTTAAGACAACAAAGAGA<br>TGCAATAGCGGAATGGAGTAGATTAACTTATGGTTGGATGGGTAGAACTCCTGATTAT<br>AAAGCAGCGTTTGGATCTGCTTTAGGTGCAAATCCCGGATTTTATGGCAGATTTGAAGA

	TAATGCCAAGACTTGGTACAAGAGAATACAAGAGGCTTGTTTATATCTTAATCACGCG<br>ATTGTAAATCCACCTATTGATCGTGATAAACCTGTTGATCAAGTGAAGGATGTCTTTAT<br>ATCAGTTGATGAAGAGGTGGATGGTGGAATTGTAGTTTCTGGTGCAAAAGTCGTGGCT<br>ACAAACTCGGCTTTAACGCATTATAATTTCGTTGGTCAAGGAAGTGCCCAGTTGTTAGG<br>TGATAATACGGATTTTGCATTAATGTTTATTGCTCCAATGAATACTCCTGGTATGAAATT<br>AATTTGTAGACCGTCTTATGAGCTTGTAGCCGGCATTGCAGGTAGTCCCTTTGATTATC<br>CTTTGAGTTCCAGATTTGACGAGAACGATGCTATTTTAGTGATGGATAAGGTCTTTATA<br>CCTTGGGAAAATGTTTTAATTTACCGTGATTTTGAAAGGTGTAAACAATGGTTTCCTCA<br>AGGCGGTTTTGGGCGTTTATTTCCTATGCAAGGGTGTACTAGGCTTGCAGTTAAATTAG<br>ATTTTATTACGGGTGCACTGTATAAAGCATTACAGTGTACTGGAAGTTTAGAATTTCGT<br>GGGGTTCAAGCCCAAGTGGGTGAGGTTGTTGCTTGGAGAAATTTGTTTTGGAGCTTAAC<br>TGATGCAATGTATGGTAATGCATCCGAGTGGCATGGTGGAGCTTTTCTTCCATCAGCTG<br>AAGCATTGCAAGCATATCGTGTTTTGGCCCCACAAGCATATCCAGAAATAAAGAAAAC<br>AATTGAACAAGTTGTAGCTTCGGGTTTGATTTATTTACCGAGCGGTGTAAGGGATCTTC<br>ATAACCCCCAGTTGGATAAATACTTGTCAACATACTGTAGAGGATCTGGTGGTATGGG<br>ACATAGAGAAAGAATTAAGATATTAAAATTGTTATGGGATGCTATAGGTTCGGAATTT<br>GGTGGTCGTCACGAACTATATGAAATAAACTATGCGGGATCACAAGATGAAATCAGAA<br>TGCAAGCATTAAGACAAGCTATTGGATCTGGTGCTATGAAAGGAATGTTGGGGATGGT<br>AGAACAATGTATGGGTGATTATGATGAAAATGGATGGACTGTTCCTCATCTTCATAATC<br>CTGATGATATTAATGTTTTGGACAGAATACGTCAGTAA
1- *Se*HpaC<br>2- *Salmonella enterica*	ATGCAAGTAGATGAACAACGTCTGCGTTTTCGCGATGCGATGGCAAGTCTGGCGGCAG<br>CGGTCAACATCGTAACCACGGCGGGTCACGCCGGACGCTGCGGTATCACCGCAACAGC<br>GGTCTGTTCCGTCACCGATACGCCGCCCTCCGTGATGGTATGTATTAATGCCAATAGCG<br>CCATGAACCCCGTCTTTCAGGGCAACGGCAAGCTGTGCATTAATGTACTTAACCATGAG<br>CAGGAGCTGATGGCGCGCCACTTTGCCGGTATGACGGGGATGGCGATGGAAGAGCGTT<br>TTCACCAGCCATGTTGGCAAAACGGGCCGCTGGGCCAGCCGGTACTTAACGGCGCGCT<br>GGCCGGTCTTGAAGGCGAGATCAGCGAGGTACAAACCATTGGCACGCATCTGGTGTAT<br>CTGGTGGCGATCAAAAATATTATTCTTAGCCAGGATGGGCATGGCCTGATTTATTTCAA<br>ACGCCGTTTTCATCCGGTCAGACTTGAGATGGAAGCGCCTGTTTAA
1- *At*COMT (opt)<br>2- *Arabidopsis thaliana*	ATGGGAAGTACCGCTGAAACTCAGCTAACGCCAGTTCAAGTAACAGATGATGAGGCCG<br>CTTTATTTGCTATGCAGCTGGCTTCAGCATCAGTCTTGCCTATGGCATTGAAGTCTGCTC<br>TTGAACTGGATTTATTAGAAATAATGGCAAAGAACGGCTCCCCAATGTCCCCAACAGA<br>AATTGCATCAAAGTTACCAACGAAGAACCCAGAGGCACCCGTCATGTTAGATAGAATT<br>TTGAGATTATTGACTTCATATTCTGTGCTTACATGTTCAAATAGAAAGTTGTCAGGAGA<br>CGGTGTGGAGAGGATCTATGGTTTGGGCCCAGTGTGTAAATACTTAACTAAGAATGAG<br>GACGGCGTCTCGATAGCAGCGTTGTGCTTGATGAATCAGGATAAAGTATTAATGGAGT<br>CCTGGTATCACCTAAAAGACGCTATCTTAGACGGCGGTATCCCGTTTAATAAAGCCTAC<br>GGTATGTCTGCTTTTGAATATCATGGAACGGACCCCAGGTTTAATAAAGTGTTCAATAA<br>CGGTATGAGCAATCACTCAACTATAACGATGAAGAAGATCTTGGAAACTTACAAAGGG<br>TTCGAGGGCTTAACCAGCTTAGTCGACGTAGGCGGCGGTATAGGCGCGACGCTGAAGA<br>TGATCGTATCTAAATATCCAAACTTAAAGGGTATTAATTTCGACTTGCCTCACGTTATT<br>GAGGACGCACCATCACACCCCGGAATAGAACACGTGGGTGGTGACATGTTCGTCTCAG<br>TACCAAAGGGCGACGCTATCTTTATGAAATGGATTTGCCACGATTGGTCTGATGAGCAC<br>TGTGTTAAGTTTCTAAAGAATTGTTATGAATCTTTGCCCGAAGACGGTAAGGTTATTCT<br>TGCTGAATGCATTCTGCCCGAAACTCCCGATTCCTCTTTAAGCACTAAGCAGGTGGTGC<br>ACGTGGACTGTATCATGCTAGCCCATAACCCAGGCGGAAAGGAGAGGACGGAAAAGG<br>AATTCGAAGCCTTGGCTAAGGCTTCTGGTTTTAAAGGTATTAAGGTGGTTTGTGATGCA<br>TTCGGAGTCAATTTAATAGAATTGTTGAAGAAACTATAG
1- *Pp*FerA (opt)<br>2- *Pseudomonas paucimobilis*	ATGACGGTCGAGGCGGGAGTTAGGCCGCAGGCCGGAGCCAGGGATATAAATAGGTTA<br>CTTAGACCCAGGTCCATAGCCATAGTTGGGGCAAGTGAAACGCCTGGTTCTTTGGGGG<br>CTTCAGTCCTAGCGAACTTAGTAAGGAATGAATTTCCGGGTGACATCCATCTGGTCAAC<br>CCTAAAAGAGAAACCATAAGTGGTAGGCCTGCAGTTCCTTCCGTCGATGCACTTCCGG<br>AAGGTGTGGATTGTGCTATACTAGCGATACCAAGAGTTGCCGTTCTAGACACTATGCGT<br>CAACTTGCAGCTAGAAAGGCTGGGGCCGCCATCATTTTTGCTGCAGGGTTTGCTGAGGG<br>TGGTGAGCAGGGAATGGCGGATCAACAAGAGATAGGGAGAATCGCCCATGAGGCCGG<br>GATAGTGGTAGAGGGTCCGAACTGCTTGGGATCTGTTAACTATTTAGACAGGATCCCGC<br>TGACATTCATCGACACCGATATCAAAGCACCCTCTCCAGGCGGAGTTGGTATAGTTAGC<br>CAGTCCGGGGCTATGGCTGCGGTACTAGCGGTGATGCTAGAGAGTAGGACGTTGGATT<br>TAACGTACAGTGTCTCCACTGGTAATGAGGCAGGTTCCGGAGTGGAAGACTATGTCGA<br>GTTCATGATCGCAGATGAGAAAACCAGAATCATCGCAATGATAGTGGAGCAGTTCAGG<br>GACCCGGCTCGTTTCTTGGCGCTAATGGACAAGGCTAATGCAGCCGGCAAGTTAGTCGT<br>ACTGTTGCATCCGGGAAAGTCTTCTGCTGCCAGAGAAAGCGCGGCGACCCATACGGGG<br>GCAATGGCTGGCGATTATAAGCTGATGAGGGCGAAAGTAGAAAGAGCCGGCGTAGTTG<br>TCGCTGAAACCTTAGAAGAACTAGGAGATATAACGGAAATCGCAGCTAGGTGCCCCGC<br>TCTACCCAGCGGTGGCACAGCCGTCCTGGGGGAATCAGGTGCACTAAAGGCACTAACG<br>TTAGACCTGGCCGAGGAACTAGAGTTGGCATTACCCACGTTGGATGACGGGAATGCGC<br>CGGCTCTTAGAGCAGCACTGCCTGAGTTCGTCCCGGTAAGTAACCCACTAGATTTGACA<br>GCGCAAGGATTAGTGGACCCAGATATGTACTACCGTACACTAGCCGCGCTATTCGAGG<br>ATGACCGTGTGGGGACGATATTCGCGGGGATCATCCAAACGAATCCGGCGACCATAGG

	TATCAAACTACCACCGTTTCTAAAAGCAGTTCGTGAGCTGAAGGCGACCAAACCAGTC<br>ATATTTGGGGGAGTCGATGAAGGAGCGGATGTACCCGCAGATTGGATAGAGCAACTTA<br>GAGCGGAAGGTATACCCTACTTTCCGACTACGGAACGTGCCTTGCGTGCCATTCGTCGT<br>CTGAGTGCGGCCGGTGCGAGGGACGCCTCCAGGACGGATGCAGCTCCCGCTTCAGTCC<br>CCGCCTTGGCTTCTGAGAAGGGGGTTGTTCCAGAGTACAAAGCAAAGGCACTGCTTGC<br>GCCCTTGGGAATATCATTCCCAAAAGGACAGTTTGCGGCAACCGTTGAAGACGCCATC<br>GCCGCTGCGGAAGCCATAGGGGGGCCGGTGGTAATGAAGGCACAAGCGGCCGCTCTAT<br>CTCATAAGAGTGATGCCGGGGGAGTTGCGTTGAACCTGGTGGGTGCTGAGGCCATCCG<br>TGCTGCATGGGATAAAATGTTCGCCGATGTGAAGAGGTATGATGCCTCCATCATACTAG<br>ACGGGGTGCTTATCGAGGCTATGGGTGCGAGAGGCTTGGAGCTAATAGTAGGAGCCAA<br>GAACGACCCACAGTGGGGTCCTGTAATCTTAGCCGGATTTGGTGGGGTGACAGCCGAA<br>ATATTGCAGGACGTGAGATTACTAAGCCCCGACATGACTAAGGAGGCCATCGTAGCTG<br>AACTTGGAAAACTGAAGCAAGCGGCATTGCTGCACGGTTATAGAGGTTCCCCAGCACT<br>AGACGTGGGCGCCGTTGCTGAACTAATAGGACAAGTGGGAAGATTGCTAAGAGGGGA<br>GCCCAGGATACAGGAACTGGATCTTAACCCGGTGGTCGTATACCCAGAAGGCCAGGGG<br>GCAATCGCACTTGATGCTCTTATGTTGGTAGATTGA
1- *Cl*DCS (opt)<br>2- *Curcuma longa*	ATGGAAGCTAACGGTTATAGAATCACTCACTCTGCCGATGGTCCAGCTACGATTTTAGC<br>TATCGGCACTGCTAACCCTACTAATGTTGTTGATCAAAATGCTTATCCAGATTTCTATTT<br>TAGAGTCACCAATTCAGAATACTTACAGGAGTTAAAGGCTAAGTTTAGAAGAATCTGT<br>GAGAAGGCCGCCATAAGAAAGCGGCATTTGTACTTGACCGAGGAAATTCTTAGAGAAA<br>ATCCAAGTTTGCTGGCTCCAATGGCGCCCAGCTTCGATGCAAGACAAGCAATTGTGGTG<br>GAAGCGGTCCCCAAACTCGCTAAGGAAGCTGCCGAGAAGGCCATTAAGGAGTGGGGT<br>AGACCTAAAAGCGACATCACGCATCTCGTTTTCTGCTCGGCATCTGGTATCGATATGCC<br>AGGTTCCGATCTGCAGTTATTAAAATTGTTGGGTCTTCCACCATCAGTCAATCGTGTCA<br>TGTTATATAACGTTGGTTGTCACGCTGGTGGTACAGCCTTGAGGGTTGCCAAAGATTTG<br>GCGGAAAATAATAGAGGCGCCCGTGTTCTGGCAGTTTGTAGCGAAGTTACTGTCCTTAG<br>TTATAGAGGTCCACACCCAGCACACATAGAATCACTTTTCGTCCAAGCCCTTTTCGGAG<br>ATGGAGCTGCCGCCTTGGTCGTCGGCTCCGACCCCGTAGACGGAGTAGAAAGACCCAT<br>ATTTGAAATCGCTTCCGCTTCACAAGTCATGTTACCCGAATCTGCTGAAGCCGTAGGAG<br>GGCACTTGAGGGAAATTGGCTTGACCTTCCATTTAAAATCTCAGTTACCATCCATTATT<br>GCGTCCAATATCGAACAATCCTTGACTACAGCCTGCTCCCCATTGGGGCTGTCAGATTG<br>GAATCAATTGTTCTGGGCCGTGCACCCTGGAGGCAGGGCCATACTTGATCAAGTGGAA<br>GCAAGATTGGGCCTTGAAAAAGATCGTCTTGCCGCGACACGGCACGTACTGTCAGAAT<br>ACGGTAACATGCAATCAGCCACAGTGTTGTTCATTTTGGATGAAATGAGAAACAGGAG<br>TGCCGCTGAGGGACACGCGACTACCGGCGAGGGATTGGATTGGGGAGTGCTGTTGGGT<br>TTTGGGCCAGGTTTGAGTATAGAAACTGTAGTCTTACATTCATGCAGGCTTAATTGA
1- *Cl*CURS1 (opt)<br>2- *Curcuma longa*	ATGGCCAATCTACATGCGCTTAGAAGGGAACAAAGAGCTCAGGGTCCAGCAACAATTA<br>TGGCTATCGGTACTGCTACTCCACCTAACTTATACGAACAGTCGACATTCCCGGACTTT<br>TATTTTCGTGTTACAAACTCAGATGATAAGCAAGAACTCAAAAAAAAGTTTAGAAGAA<br>TGTGCGAAAAGACAATGGTGAAGAAGCGTTACCTGCATCTAACAGAGGAAATATTAAA<br>GGAGCGTCCTAAATTATGTTCTTACAAAGAAGCCAGTTTCGACGATAGACAAGATATT<br>GTGGTTGAAGAAATTCCACGTTTGGCTAAGGAAGCTGCTGAAAAGGCAATTAAAGAAT<br>GGGGTCGGCCAAAATCTGAAATTACACATCTTGTCTTCTGTTCAATAAGTGGTATTGAC<br>ATGCCAGGTGCGGACTACAGGTTAGCCACTTTGCTGGGCTTACCTCTTACAGTTAATCG<br>TCTTATGATTTATAGTCAGGCCTGTCACATGGGTGCTGCTATGTTGCGAATAGCAAAAG<br>ATTTGGCTGAGAACAACAGAGGCGCCCGCGTATTGGTTGTGGCGTGCGAAATCACAGT<br>TCTGTCTTTCAGAGGTCCAAACGAGGGAGATTTTGAAGCATTAGCTGGACAAGCGGGC<br>TTTGGCGACGGGGCCGGCGCGGTCGTTGTAGGTGCCGACCCGCTCGAAGGTATAGAAA<br>AACCAATCTACGAAATCGCTGCTGCAATGCAGGAAACAGTGGCAGAATCACAGGGTGC<br>AGTGGGGGGCCATTTGAGGGCTTTTGGATGGACCTTTTATTTCCTTAACCAGTTACCAG<br>CAATTATTGCTGACAATTTGGGTAGAAGCCTTGAACGAGCACTTGCCCCACTCGGTGTC<br>CGCGAATGGAATGACGTTTTTTGGGTGGCCCATCCTGGTAACTGGGCAATTATTGATGC<br>TATCGAAGCAAAGTTGCAATTAAGTCCCGATAAATTGAGTACCGCAAGACATGTTTTCA<br>CTGAATACGGTAACATGCAGAGTGCAACCGTTTACTTTGTAATGGATGAATTACGAAA<br>AAGATCTGCCGTGGAGGGTAGATCAACAACTGGAGATGGTCTGCAATGGGGTGTTCTG<br>TTGGGGTTTGGCCCAGGATTGTCAATTGAAACCGTGGTTCTAAGGTCCATGCCCTTGTA<br>A
1- ACC1<br>2- *Saccharomyces cerevisiae*	SGD systematic name: YNR016C
1- FOX1<br>2- *Saccharomyces cerevisiae*	SGD systematic name: YGL205W

Table S3: Plasmids used in this work.

Plasmids	Features	Reference
pSP-GM1	AmpR, URA3, ori, 2µ ori	Addgene plasmid: #64739
pBEVY-GL	AmpR, LEU, ori, 2µ ori	Addgene plasmid: #51225
pSP-GM1_FerA_DCS_CURS	pSPGM1; p_{TEF1}-*Cl*DCS-t_{ADH1}; p_{TDH3}-*Cl*CURS1-t_{synt27}; p_{PGK1}-*Pp*FerA-t_{CYC1}	[1]
pSP-GM1_HpaC	pSPGM1; p_{TEF1}-*Se*HpaC-t_{ADH1}	This study
pSP-GM1_HpaB_HpaC	pSPGM1_*Se*HpaC; p_{PGK1}-*Pp*HpaB-t_{CYC1}	
pSP-GM1_HpaBopt_HpaC	pSPGM1_*Se*HpaC; p_{PGK1}-*Pp*HpaBopt-t_{CYC1}	
pSP-GM1_COMT	pSPGM1; p_{TEF1}-*At*COMT-t_{ADH1};	
pBEVY-GL_DCS	pBEVY-GL; p_{GAL10}-*Cl*DCS-t_{ADH1}	
pBEVY-GL_DCS_CURS	pBEVY-GL_DCS; p_{GAL1}-*Cl*CURS1-t_{ADH2}	
pBEVY-GL_FerA	pBEVY-GL; p_{GAL1}-*Pp*FerA-t_{ADH2}	
pSP-GM1_FOX	pSP-GM1; p_{PGK1}-FOX1-t_{CYC1}	
pSP-GM1_ACC	pSP-GM1; p_{TEF1}-ACC1-t_{ADH1}	
pSP-GM1_FOX1_ACC	pSP-GM1_ACC; p_{PGK1}-FOX1-t_{CYC1}	
pLC-C3	AmpR, HIS3, ori, 2µ ori, p_{PGK1}-coC3H-t_{CYC1}, p_{TEF1}-coCPR1-t_{ADH1}	[2]
gRNA plasmids		
pQLC09	2µ ori, AmpR, KlURA3, gRNA-XI-5.Y	[3]
pQLC10	2µ ori, AmpR, KlURA3, gRNA-XII-4.Y	
pQLC133	2µ ori, AmpR, URA3, gRNA-XII-3.Y	
pQLC130	2µ ori, AmpR URA3 gRNA-XI-2.Y	
pQLC032	2µ ori, AmpR URA3, gRNA-XII-1.Y	

Table S4: *Saccharomyces cerevisiae* strains used in this study.

Strains	Genotype	Reference
IMX581	MATa ura3-52 can1D::cas9-natNT2 TRP1 LEU2 HIS3	Euroscarf: Y40593
QL158	IMX581, pdc5Δ, aro10Δ, XII-2::(p$_{GPM1}$-*At*PAL2-t$_{FBA1}$)+(p$_{TDH3}$-*At*C4H-t$_{CYC1}$)+(p$_{HXT7}$-*At*ATR2-t$_{YX212}$)+(p$_{PGK1}$-CYB5-t$_{ADH1}$), X-3::(p$_{TPI1}$-*Ec*aroL-t$_{YX212}$)+(t$_{ADH1}$-ARO7 G141S-p$_{TEF1}$)+(p$_{PGK1}$-ARO4 K229L-CYC1t), X-4::(t$_{CYC1}$-ARO1-p$_{TPI1}$)+(p$_{TDH3}$-ARO2-t$_{ADH1}$)+(t$_{TDH2}$-ARO3-p$_{TEF1}$), XI-3::(p$_{TEF1}$-*Fj*TAL-t$_{TDH2}$)+(t$_{FBA1}$-*Mt*PDH1-p$_{TDH3}$)	[3]
JI1	IMX581, XII-4::(p$_{TEF1}$-*Cl*DCS-t$_{ADH1}$; p$_{TDH3}$-*Cl*CURS-t$_{synt27}$; p$_{PGK1}$-*Pp*FerA-t$_{CYC1}$), pQLC10	This study
JI2	JI1, XII-1::(p$_{TEF1}$-*At*COMT-t$_{ADH1}$), pQLC032	
JI3	JI2, XI-5::(p$_{TEF1}$-*Se*HpaC-t$_{ADH1}$; p$_{PGK1}$-*Pp*HpaB-t$_{CYC1}$), pQLC09	
JG0	IMX581, XII-4::(p$_{GAL1}$-*Cl*DCS-t$_{ADH1}$; p$_{GAL10}$-*Cl*CURS-t$_{ADH2}$), pQLC10	
JG1	JG0, XII-3::(p$_{GAL10}$-*Pp*FerA-T$_{ADH2}$), pQLC133	
JQ01	QL158, XII-4::(p$_{TEF1}$-*Cl*DCS-t$_{ADH1}$; p$_{TDH3}$-*Cl*CURS-t$_{synt27}$; p$_{PGK1}$-*Pp*FerA-t$_{CYC1}$), pQLC10	
JQ02	JQ01, XII-1::(p$_{TEF1}$-*At*COMT-t$_{ADH1}$), pQLC032	
JQ1	JQ02, XI-5::(p$_{TEF1}$-*Se*HpaC-t$_{ADH1}$;p$_{PGK1}$-*Pp*HpaB-t$_{CYC1}$), pQLC09	
JQCA1	QL158, XI-5::(p$_{TEF1}$-*Se*HpaC-t$_{ADH1}$;p$_{PGK1}$-*Pp*HpaB-t$_{CYC1}$), pQLC09	
JQCA2	QL158, XI-5::(p$_{TEF1}$- coC3H-t$_{ADH1}$;p$_{PGK1}$-coCPR1-t$_{CYC1}$), pQLC09	
JQCA3	QL158, XI-5::(p$_{TEF1}$-*Se*HpaC-t$_{ADH1}$;p$_{PGK1}$-*Pp*HpaB(opt)-t$_{CYC1}$), pQLC09	
JQ2	JQ02, XI-5::(p$_{TEF1}$-*Se*HpaC-t$_{ADH1}$;p$_{PGK1}$-*Pp*HpaB(opt)-t$_{CYC1}$), pQLC09	
JQ3	JQ2, XII-3::(p$_{TEF1}$-*Se*HpaC-t$_{ADH1}$; p$_{PGK1}$-*Pp*HpaB-t$_{CYC1}$), pQLC133	
JQ4	JQ3, XI-2::(p$_{TEF1}$-*At*COMT-t$_{ADH1}$), pQLC130	

Table S5: Primers used in this work. For gene amplification primers, the bold sequence represents the restriction enzyme sequence used to clone the gene, and the underlined sequence represents the homology sequence for cloning via assembly cloning. For cassette amplification primers, the underlined sequence represents the 25-bp homology sequence for the corresponding homology arms.

Primer name	Sequence 5'-3'
Gene amplification	
HpaB_FW	GC**GGATCC**AAAAAAATGAAACCAGAAGACTTTAGA (BamHI)
HpaB_RV	AACCC**AAGCTT**TTACTGACGTATTCTGTCCA (HindIII)
HpaC_FW	AAAAA**GAGCTC**TTAAACAGGCGCTTCCATC (SacI)
HpaC_RV	A**GCGGCCGC**AAAAAAATGCAAGTAGATGAACAACG (NotI)
HpaBopt_FW	CGTAATACGACTCACTATAGGGCCCAAAAAAATGAAACCAGAAGACTTTAGA
HpaBopt_RV	CAACTTCTGTTCCATGTCGACGCCCTTACTGACGTATTCTGTCCA
COMT_FW	TTTTC**GAGCTC**CTATAGTTTCTTCAACAATTCTATTAA (SacI)
COMT_RV	AAAA**GCGGCCGC**AAAAAAATGGGAAGTACCGCTGAA (NotI)
DCS_GAL_FW	AAAA**TCTAGA**TCAATTAAGCCTGCATGAA (XbaI)
DCS_GAL_RV	AAAA**GGATCC**AAAAAAATGGAAGCTAACGG (BamHI)
CURS_GAL_FW	AAAA**GGTACC**AAAAAAATGGCCAATCTACAT (KpnI)
CURS_GAL_RV	AAAA**GAATTC**TTACAAGGGCATGGACCTTA (EcoRI)
FerA_GAL_FW	AAAA**GGTACC**AAAAAAATGGCCAATCTACAT (KpnI)
FerA_GAL_RV	AAAA**GAATTC**TCAATCTACCAACATAAGAG (EcoRI)
ACC_FW	AAA**GAGCTC**TTATTTCAAAGTCTTCAACAATTT (SacI)
ACC_RV	T**GCGGCCGC**AAAAAAATGAGCGAAGAAAGCTTATT (NotI)
FOX_FW	CGTAATACGACTCACTATAGGGCCCAAAAAAATGACGAGACGTACTACTATTAA
FOX_RV	CAACTTCTGTTCCATGTCGACGCCCTCACTTGTTTATTTCGATAAAAT
Colony PCR	
pPGK1_FW	GTGATCTCCAGAGCAAAGT
tCYC_RV	CTTCGAGCGTCCCAAAAC
tAHD1_FW	GTCACTTTAAAATTTGTATAC
pTEF_RV	CGTACCACTTCAAAACACC
pGAL_FW	CAACCATAGGATGATAATGCGAT
pGAL_RV	TTTACATTTCCACAACATATAAGTAAG
tADH2_RV	TTATTCGTGAAACTTCGAACAC
Homology arms amplification	
XII-4_US_FW	GTATCCGGCTGTTCCTTCATA
XII-4_US_RV	TGCCATAGTATGTGTGATGGAA
XII-4_DS_FW	ATTCCCCATTAGAGTCAAATAAA
XII-4_DS_RV	TTTCTGCCGTACCTGGAT
XI-5_US_FW	GCGGAGAAGTCGTTGATAG
XI-5_US_RV	TGGTGCACGGAGTTTATGG
XI-5_DS_FW	GCCCACAACCCAAGTTAA
XI-5_DS_RV	GATCATAGATCCGGCACTT
XI-2_US_FW	TAACTCTTCGTATGAGGATTTTC
XI-2_US_RV	TTCTATGGCACATTTTTCTGT
XI-2_DS_FW	CCACAAGTAAAGCTCGTTGA
XI-2_DS_RV	ATGGTTGAAAAGGTTACAGAG
XII-1_US_FW	GTTGAGCTCTGTCCTTCATG
XII-1_US_RV	GAAAGAACCGAACCGATGC
XII-1_DS_FW	GTTCAGTTTAGTGCTCTGT
XII-1_DS_RV	TGATGACTGTTTCTCAATCTTT
XII-3_US_FW	TGTGCCCCTTAAAATTCATATAC
XII-3_US_RV	GAATGAGCAGGTACCCCTTAT
XII-3_DS_FW	GCATAGAGCTAATTAGGTTTGAG
XII-3_DS_RV	GAACTTACAAGCTGATTTTGGTTC

Cassette amplification	
XII-4_pTDH3_FW	TTTTTCCATCACACATACTATGGCACTATTTCGAGGACCTTGTCACC
XII-4_tCYC1_RV	CTTTTATTTGACTCTAATGGGGAATCTTCGAGCGTCCCAAAACC
XI-5_tADH1_FW	AATGTGCCATAAACTCCGTGCACCAGAGCGACCTCATGCTATACC
XI-5_tCYC1_RV	CTTATGATTAACTTGGGTTGTGGGCCTTCGAGCGTCCCAAAACC
XI-2_tADH1_FW	CTCAACAGAAAAATGTGCCATAGAAGAGCGACCTCATGCTATACCTG
XI-2_pTEF1_RV	ACTGGTCAACGAGCTTTACTTGTGGGCACACACCATAGCTTCAAAAT
XII-1_tADH1_FW	ATTTTGGCATCGGTTCGGTTCTTTC GAGCGACCTCATGCTATACCTG
XII-1_pTEF1_RV	ACTCAGACAGAGCACTAAACTGAACGCACACACCATAGCTTCAAAAT
XII-3_tADH1_FW	TATAATAAGGGGTACCTGCTCATTCGAGCGACCTCATGCTATACCTG
XII-3_tCYC1_RV	AACTCAAACCTAATTAGCTCTATGCCTTCGAGCGTCCCAAAACC
XII-4_tADH1_FW	TTTTTCCATCACACATACTATGGCA CCGGTAGAGGTGTGGTCA
XII-4_tADH2 _RV	CTTTTATTTGACTCTAATGGGGAATATGAGAAATATCGAGGGAGAC
XII-1_pGAL_FW	ATTTTGGCATCGGTTCGGTTCTTTCTTTCAAAAATTCTTACTTTTTTTT
XII-1_tADH2_RV	ACTCAGACAGAGCACTAAACTGAACATGAGAAATATCGAGGGAGA

References

[1] Rainha, J., Rodrigues, J. L., Faria, C., & Rodrigues, L. R. (2022). Curcumin biosynthesis from ferulic acid by engineered *Saccharomyces cerevisiae*. *Biotechnology Journal*, 17(3), 2100400.

[2] Li, Y., Mao, J., Liu, Q., Song, X., Wu, Y., Cai, M., ... & Qiao, M. (2020). *De novo* biosynthesis of caffeic acid from glucose by engineered *Saccharomyces cerevisiae*. *ACS Synthetic Biology*, 9(4), 756-765.

[3] Liu, Q., Yu, T., Li, X., Chen, Y., Campbell, K., Nielsen, J., & Chen, Y. (2019). Rewiring carbon metabolism in yeast for high level production of aromatic chemicals. *Nature communications*, 10(1), 4976.
